# Retraction Note: Upregulation of OSBPL3 by HIF1A promotes colorectal cancer progression through activation of RAS signaling pathway

**DOI:** 10.1038/s41419-022-05367-7

**Published:** 2022-11-02

**Authors:** Hong-li Jiao, Bin-shu Weng, Shan-shan Yan, Zi-mo Lin, Shu-yang Wang, Xiao-ping Chen, Guang-hua Liang, Xiao-Qing Li, Wei-yi Zhao, Jia-Yi Huang, Dan Zhang, Ling-jie Zhang, Fang-yi Han, Sheng-nan Li, Li-jie Chen, Jiong-hua Zhu, Wen-feng He, Yan-qing Ding, Ya-ping Ye

**Affiliations:** 1 Department of Pathology, Nanfang Hospital, Southern Medical University, Guangzhou, Guangdong China; 2 Department of Pathology, School of Basic Medical Sciences, Southern Medical University, Guangzhou, Guangdong China; 3 Guangdong Provincial Key Laboratory of Molecular Tumor Pathology, Guangzhou, Guangdong China; 4 Department of Pathology, Guangdong Women and Children Hospital, Guangzhou, China

Retraction to: *Cell Death and Disease* 10.1038/s41419-020-02793-3, published online 24 July 2020

The Editors-in-Chief have retracted this article. After publication, concerns were raised regarding suspected image duplication and overlap. Specifically:

- In Fig. 3a, the cell growth curves for the two cell lines appear highly similar;
- In Fig. 3e, the H&E images for shRNA#1 and shRNA#2 appear to overlap;
- In Fig. 3h, the intestine images in the scramble and shRNA#1 groups appear to originate from the same sample;
- Also in Fig. 3h, the liver images in the shRNA#1 and shRNA#2 groups appear highly similar (rotated 90 degrees with brightness adjustment);
- In Fig. 4c HCT116 shRNA data, the bands representing OSBPL3 and P27 appear highly similar (rotated 180 degrees);
- In Fig. 5a, the HCT15 OSBPL3 bands appear highly similar to the HCT166 OSBPL3 and P27 blots in Fig. 4c;
- In Fig. S2g, the image representing the HCT15 vector group appears highly similar to Fig. S6e RKO HIF1a+OSBPL3-shRNA#1;
- In Fig. S3f, the images for HCT116 shRNA#1 and shRNA#2 appear to overlap.
- In Table 1, the total patient numbers in the low and high OSBPL3 expression groups are inconsistent.

The Editors-in-Chief therefore no longer have confidence in the presented data.

None of the authors have responded to any correspondence from the editor or publisher about this retraction.

